# A familial case of *MYH9* gene mutation associated with multiple functional and structural platelet abnormalities

**DOI:** 10.1038/s41598-022-24098-5

**Published:** 2022-11-20

**Authors:** Svetlana I. Safiullina, Natalia G. Evtugina, Izabella A. Andrianova, Rafael R. Khismatullin, Olga A. Kravtsova, Alina I. Khabirova, Chandrasekaran Nagaswami, Amina G. Daminova, Alina D. Peshkova, Rustem I. Litvinov, John W. Weisel

**Affiliations:** 1 Institute of Fundamental Medicine and Biology, Kazan Federal University, Kazan, Russian Federation; 2 Department of Cell and Developmental Biology, University of Pennsylvania School of Medicine, 421 Curie Blvd., BRB II/III, Room 1154, Philadelphia, PA 19104-6058 USA

**Keywords:** Cardiovascular diseases, Haematological diseases

## Abstract

Mutations in the *MYH9* gene result in macrothrombocytopenia often associated with hemorrhages. Here, we studied the function and structure of platelets in three family members with a heterozygous mutation R1933X in the *MYH9* gene, characteristic of closely related disorders known as the May-Hegglin anomaly and Sebastian syndrome. The examination included complete blood count, blood smear microscopy, platelet flow cytometry (expression of P-selectin and active integrin αIIbβ3 before and after activation), the kinetics of platelet-driven contraction (retraction) of blood clots, as well as scanning/transmission electron microscopy of platelets. Despite severe thrombocytopenia ranging (36–86) × 10^9^/l, none of the patients had hemorrhages at the time of examination, although they had a history of heavy menstruation, spontaneous ecchymosis, and postpartum hemorrhage. Flow cytometry showed background platelet activation, revealed by overexpression of P-selectin and active αIIbβ3 integrin above normal levels. After TRAP-induced stimulation, the fractions of platelets expressing P-selectin in the proband and her sister were below normal response, indicating partial platelet refractoriness. The initiation of clot contraction was delayed. Electron microscopy revealed giant platelets with multiple filopodia and fusion of α-granules with dilated open canalicular system, containing filamentous and vesicular inclusions. The novel concept implies that the R1933X mutation in the *MYH9* gene is associated not only with thrombocytopenia, but also with qualitative structural and functional defects in platelets. Platelet dysfunction includes impaired contractility, which can disrupt the compaction of hemostatic clots, making the clots weak and permeable, therefore predisposing patients with *MYH9* gene mutations to the hemorrhagic phenotype.

## Introduction

Thrombocytopenias, congenital or acquired, are characterized by a persistent decrease in platelet counts in the blood in combination with hemorrhagic disorders, having variable clinical manifestations and severity. Such quantitative changes in the number of platelets may be associated with qualitative platelet defects, i.e., thrombocytopathies. Primary genetic thrombocytopenias and thrombocytopathies, which are quite different in their underlying mechanisms and clinical phenotypes, include Bernard Soulier syndrome, Glanzmann thrombasthenia, Hermansky-Pudlak syndrome, thrombocytopenia with absent radii syndrome, grey platelet syndrome, Chediak-Higashi syndrome, Wiskott-Aldrich syndrome, storage pool deficiencies, and others^1,2^.

Among congenital thrombocytopenias and thrombocytopathies, there is a group of diseases caused by a defect in the *MYH9* gene, located in the chromosome 22q12–13 and encoding the heavy chain of non-muscle myosin IIA^3^. Myosin IIA is expressed in many cell types, but in platelets it is the main isoform of this motor protein, which, in combination with actin, generates mechanical force via an ATP-dependent contractile mechanism^4^. This force is necessary for rearrangement of the cytoskeleton while platelets perform various dynamic functions, such as spreading and aggregation, secretion of biologically active substances, as well as contraction of blood clots and thrombi^5^. Mutations in the *MYH9* gene in most cases are inherited as an autosomal dominant trait and cause a group of *MYH9-*related disorders^6^ with an overall prevalence of about 3 cases per 1,000,000 in the general population^7,8^.

Defects in the *MYH9* gene can manifest with a hemorrhagic syndrome, sometimes in combination with other pathological symptoms, such as hearing loss, kidney disease, and cataracts^3^. The cause of bleeding in both humans and mice harboring null and loss-of-function mutations in *MYH9* is the net effect of reduced platelet counts and functional anomalies. Despite many papers on the morphology of platelets in *MYH9*-related disorders, their functionality remains poorly characterized. We are aware of only a few studies in which mutations in *MYH9* caused major platelet functional defects. In a mouse model of point mutations in the *MYH9* gene, lower platelet adhesion, intercellular interaction, and traction forces were revealed^9^. Reduced contractility was determined for individual platelets in patients with a *MYH9*-related disorder^10^.

It should be noted that usually hemorrhage in the heterozygous *MYH9-*related clinical disorders is either absent or expressed slightly, with rare cases of severe bleeding, perhaps due to the heterozygous nature of mutations and compensatory hemostatic mechanisms that counteract platelet deficiency. The pathognomonic laboratory symptoms of *MYH9*-related disorders are macrothrombocytopenia (a decrease in platelet counts with a simultaneous increase in their size) in combination with the so-called Döhle-like bodies (basophilic cytoplasmic inclusions in neutrophils, monocytes and eosinophils, which comprise aggregates of a mutated heavy chain of myosin IIA)^11,12^. The mechanism of giant platelet formation (platelet macrocytosis) in *MYH9*-related disorders can be described briefly as follows. Mutations in the *MYH9* gene are associated with alterations of non-muscle myosin IIA, the main generator of contractile force in many cell types, including platelets. As soon as megakaryocytes mature, they migrate to the area of the sinusoids of bone marrow, extend protrusions into sinusoids, but the last step in the formation of platelets in the sinusoids of the bone marrow is disrupted in mutations of *MYH9*. This step depends on the combination of hemodynamic shear stress and mechanical contraction of the cytoskeleton with the participation of non-muscle myosin IIA^13,14^.

Although not listed in ICD-11, the traditional eponymous names of *MYH9*-related disorders are used broadly in the literature to specify a syndromic picture that includes the particular mutations in the *MYH9* gene associated with thrombocytopenia, platelet macrocytosis, and certain pathological phenotypic manifestation (Table S1). To the best of our knowledge, less than 1000 cases of the May-Hegglin anomaly and no more than 100 cases of the Sebastian syndrome have been described in the literature. Therefore, analysis of new cases of these rare disorders has both scientific and practical importance, especially as new methodologies of analysis become available.

The goal of the study was to get detailed information about functional and morphological changes in platelets in the family members with a documented mutation of the *MYH9* gene. Using flow cytometry, scanning and transmission electron microscopy, as well as an original clot contraction assay, it has been shown that platelets undergo background activation followed by partial refractoriness in response to stimulation, including impaired contractility. These findings indicate multiple functional and structural platelet abnormalities that can contribute to the bleeding phenotype in the May-Hegglin syndrome.

## Results

### Clinical information

The aim of this study is the examination of qualitative platelet defects induced by a *MYH9* gene mutation observed in a familial case that includes three members of the same family with an identical genotype but variable phenotypic manifestations, suggesting (epi)genetic and modifier effects.

*Patient M (proband)*, born in 1986. Menarche at the age of 14, menstruations have been profuse and painful. In adolescence, periodic spontaneous nosebleeds that stopped on their own occurred; ecchymosis on the body and limbs was also observed at the slightest traumatization. At the age of 29, thrombocytopenia, 50 × 10^9^/l, was detected accidentally for the first time. The first pregnancy at the age of 34 proceeded without hemorrhagic syndrome. Scheduled delivery at 38 weeks occurred by caesarean section. During the operation, transfusion of a platelet concentrate (3 units) was performed to manage the hemorrhagic syndrome (petechial rashes on the visceral peritoneum of the uterus and vesicouterine fold in the abdominal cavity). A girl weighing 2200 g was born. There was no postoperative bleeding. The surgical wound healed by primary intention. Because of the suspected thrombocytopathy, a blood smear was examined and genotyping was performed, which confirmed the *MYH9* gene mutation in the proband M. At the time of examination (January 2021), there were no manifestations of hemorrhagic syndrome, as well as no signs of hearing loss, defects of vision and kidney function. A newborn girl (October 2021) had a combination of thrombocytopenia (10 × 10^9^/l) and platelet macrocytosis as well as Döhle-like bodies in leukocytes, albeit without hemorrhagic syndrome.

*The proband's sister, A,* born in 1983. Menarche at 14, menstruations have been moderate and painless. At the age of 24, hemorrhagic syndrome first appeared (petechiae on the lower extremities and thrombocytopenia at 28 × 10^9^/l). After 2 courses of treatment with oral prednisolone, due to the lack of normalization of platelet counts, splenectomy was performed, which also had no positive effects, either clinical or laboratory. The first pregnancy at the age of 36 ended with an emergency caesarean section at 28 weeks due to the premature placental abruption. The operation was accompanied by bleeding and required transfusion of blood components for hemostasis. The weight of the newborn girl was 1900 g. The blood smear from the proband’s sister A, in combination with genotyping confirmed the same mutation of the *MYH9* gene revealed in the proband M. At the time of examination (January 2021), there was no hemorrhagic syndrome as well as no signs of defective hearing, vision, and kidney function. The child (proband's niece) has developed normally. No abnormalities were observed in the child's hemogram (May 2021).

*Mother of the proband, F*, born in 1959. Menarche at the age of 14, menstruations were profuse, lasting 6–7 days. She denies any other manifestations of hemorrhagic syndrome in the past, including the postoperative periods following surgical interventions (appendectomy, removal of a cyst of the maxillary sinus, myomectomy) and extraction of teeth. Two pregnancies ended in childbirth without hemorrhagic syndrome (weights of the daughters at birth were 2900 g and 2800 g). Thrombocytopenia, 86 × 10^9^/l, was revealed for the first time at the age of 57, during blood testing due to abdominal pain. At the time of the examination (January 2021), the patient had no signs of hemorrhagic syndrome, as well as no signs of hearing loss, vision defects, or kidney dysfunction.

In summary, according to the International Society on Thrombosis and Haemostasis Bleeding Assessment Tool, the proband M has a score of 4 (nosebleeds and ecchymoses—score 1 for each and heavy menstruations—score 2); the proband’s sister A has a score of 7 (nosebleeds and ecchymoses—score 1 for each, heavy menstruations—score 2, postpartum hemorrhage required transfusion of blood components—score 3); and the proband’s mother F has a score of 2 (heavy menstruations). Therefore, the proband M and her sister A were at a high risk of an inherent hemorrhagic disease (score > 3), while the proband’s mother F was at a low risk (score < 3).

*Family history of bleeding disorders.* According to the proband and her living relatives, there were no visible manifestations of hemorrhagic syndrome in the family history (Fig. S1).

### Genetic mutations

In the DNA samples of all 3 patients examined, a heterozygous nonsense mutation C5797T (R1933X) was detected in the 41st exon of the *MYH9* gene. This mutation is known to cause the formation of a premature stop-codon in mRNA, resulting in abortive transcription of the heavy chain of non-muscle myosin IIA and qualitative rather than quantitative defects of this motor protein^15,16^. As a result of this mutation, non-muscle myosin IIA is truncated by 27 amino acid residues and has a shorter non-helical tail. This region regulates filament formation through protein–protein interactions and/or phosphorylation. The R1933X mutation leads to the absence of one of the three key phosphorylation sites (Ser1943) that are important for incorporation into thick filaments and the organization of non-muscle myosin IIA assemblies. No mutations were found in the other exons of the *MYH9* gene studied. Fig. S1 provides information available on the pedigree of the patients examined and their relatives and indicates the level of platelets in their blood at the time of examination. The *MYH9* gene has bi-allelic expression and each megakaryocyte has both wild-type and mutant forms of the encoded polypeptide. However, the penetrance and expressivity of the gene depend on many environmental and genetic conditions, resulting in diverse phenotypes in the family members having the same genetic defect as revealed in our study.

### Hematologic parameters

Blood cell counts showed a decrease in the platelet counts and an increase in the immature platelet fraction in all the patients examined (Table 1). The high immature platelet fraction is characteristic of *MYH9* disorders^17^ and helps to distinguish between hereditary macrothrombocytopenias and acquired immune thrombocytopenias^18^. In addition, the proband M had an increased red blood cell (RBC) distribution width, an indicator of erythrocyte heterogeneity, in combination with lymphopenia and increased neutrophils, compared to the reference values. The proband’s sister A and the mother of the proband F had increased monocyte counts. Thus, thrombocytopenia was characteristic for all the family members examined. In addition to the proband, her sister and their mother, severe thrombocytopenia (10 × 10^9^/l) was found in the newborn daughter of the proband and moderate thrombocytopenia (178 × 10^9^/l) was revealed in the grandmother of the proband, albeit without hemorrhagic manifestations in either of them (Fig. S1).

**Table 1:** Hemogram in the patients with a *MYH9* gene mutation examined.

Parameters (in brackets—reference values)	Proband M	Sister of proband A	Mother of proband F
Platelet count (180–450), × 10^9^/l	**36**	**55**	**83**
Immature platelet fraction (1.1–6.1), %	**68.9**	**79.2**	**40.6**
Immature platelet fraction (2.5–17.8), × 10^9^/l	**24.8**	**43.6**	**33.7**
Mean platelet volume* (7.4–10.4), fl	**19.2**	**28.0**	**15.6**
Plateletcrit** (0.13–0.43), %	**0.07**	0.15	0.13
Hemoglobin (120–140), g/l	121	122	136
Hematocrit (35–45), %	35	37	39
Red blood cell count (3.7–4.7), × 10^12^/l	4.2	4.2	4.4
Mean corpuscular volume (80–100), fl	82.3	88.1	89.1
Mean corpuscular hemoglobin (27–32), pg	28.9	29.0	30.9
Mean corpuscular hemoglobin content (32–37), %	35.1	32.9	34.7
RBC distribution width (11.5–14.0), %	**15.3**	13.2	12.7
Erythrocyte sedimentation rate (2–30), mm/h	6	9	21
White blood cell (4–9), × 10^9^/l	6.3	5.5	7.2
Monocytes (2–9), %	8	**11**	**10**
Monocytes (0.08–0.81), × 10^9^/l	0.5	0.61	0.7
Lymphocytes (19–37), %	**14**	34	32
Lymphocytes (0.8–3.3), × 10^9^/l	0.9	1.9	2.3
Neutrophils (47–72), %	**77**	49	52
Neutrophils (1.9–6.5), × 10^9^/l	4.9	2.8	3.9
Eosinophils (0–5), %	0	2	3
Eosinophils (0.02–0.45), × 10^9^/l	0.02	0.11	0.18
Basophils (0–2), %	0	2	1
Basophils (0–0.12), × 10^9^/l	0.02	0.08	0.08

The numbers in bold are outside the corresponding reference range.

*Calculated manually based on the size distributions of platelets (Fig. 3D–F).

**Calculated manually using the individual mean platelet volumes and platelet counts.

### Light microscopy of blood cells

**Figure 1:**
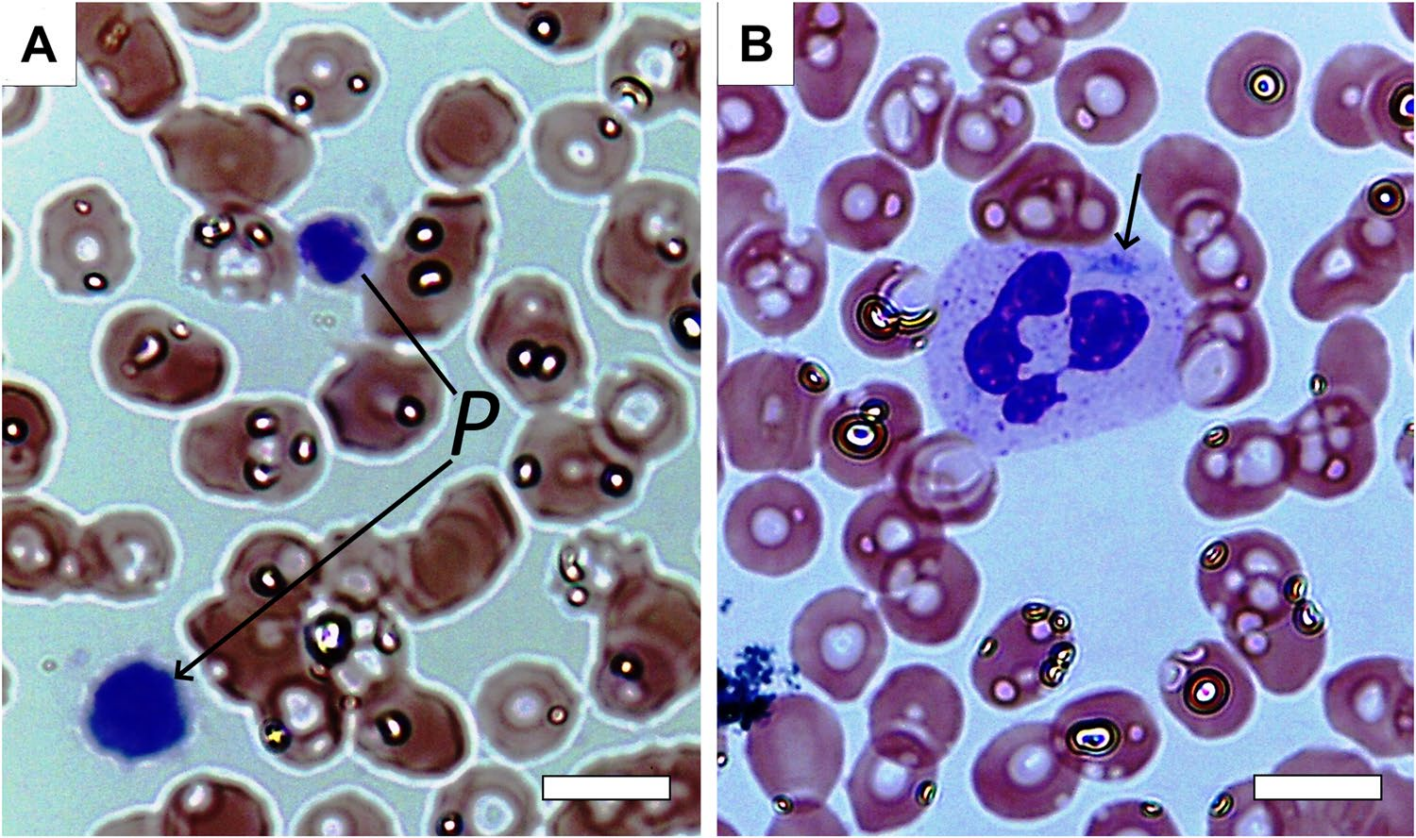
Characteristic micrographs of a blood smear from a patient with a *MYH9* gene mutation examined. (**A**) Two giant platelets (*P*) against the background of erythrocytes with slight anisocytosis and poikilocytosis. (**B**) A segmented nuclear granulocyte with a light blue fusiform cytoplasmic inclusion, a Döhle-like body (shown by the arrow). Romanowsky-Giemsa staining. Magnification bars = 6 µm.

The peripheral blood smears of the proband, her sister, and mother contained large and giant platelets, counting from 2 to 20 in each of the 10 microscopic fields studied for each patient’s smear (Fig. 1A). This corresponded to a significant increase in the average mean platelet volume in all three patients (Table 1). The mean platelet volume was calculated manually based on the size distributions of platelets because the automatic analyzer (Sysmex XN) failed to determine this parameter, due to pronounced size heterogeneity of the platelet population. White blood cells were represented by neutrophils and lymphocytes, as well as single monocytes; the number of leukocytes was within the normal range. The most remarkable finding was the presence of light blue spindle-shaped or irregularly shaped cytoplasmic inclusions often adjacent to the cell membrane (Döhle-like bodies) found in segmented leukocytes comprising neutrophils (Fig. 1B). The morphological changes revealed in leukocytes combined with macrothrombocytopenia (Table 1) and family relationship of the three patients examined corresponded to common features of *MYH9*-related disorders. The RBCs were predominantly normal-sized biconcave cells with slight anisocytosis and poikilocytosis in the form of single echinocytes and ovalocytes (Fig. 1). Despite the signs of anisocytosis of RBC observed in blood smears, the mean corpuscular volume was normal in all three patients, while in the proband M, the RBC distribution width was increased relative to the reference values (Table 1).

### Contraction of blood clots and hemostasis

**Figure 2:**
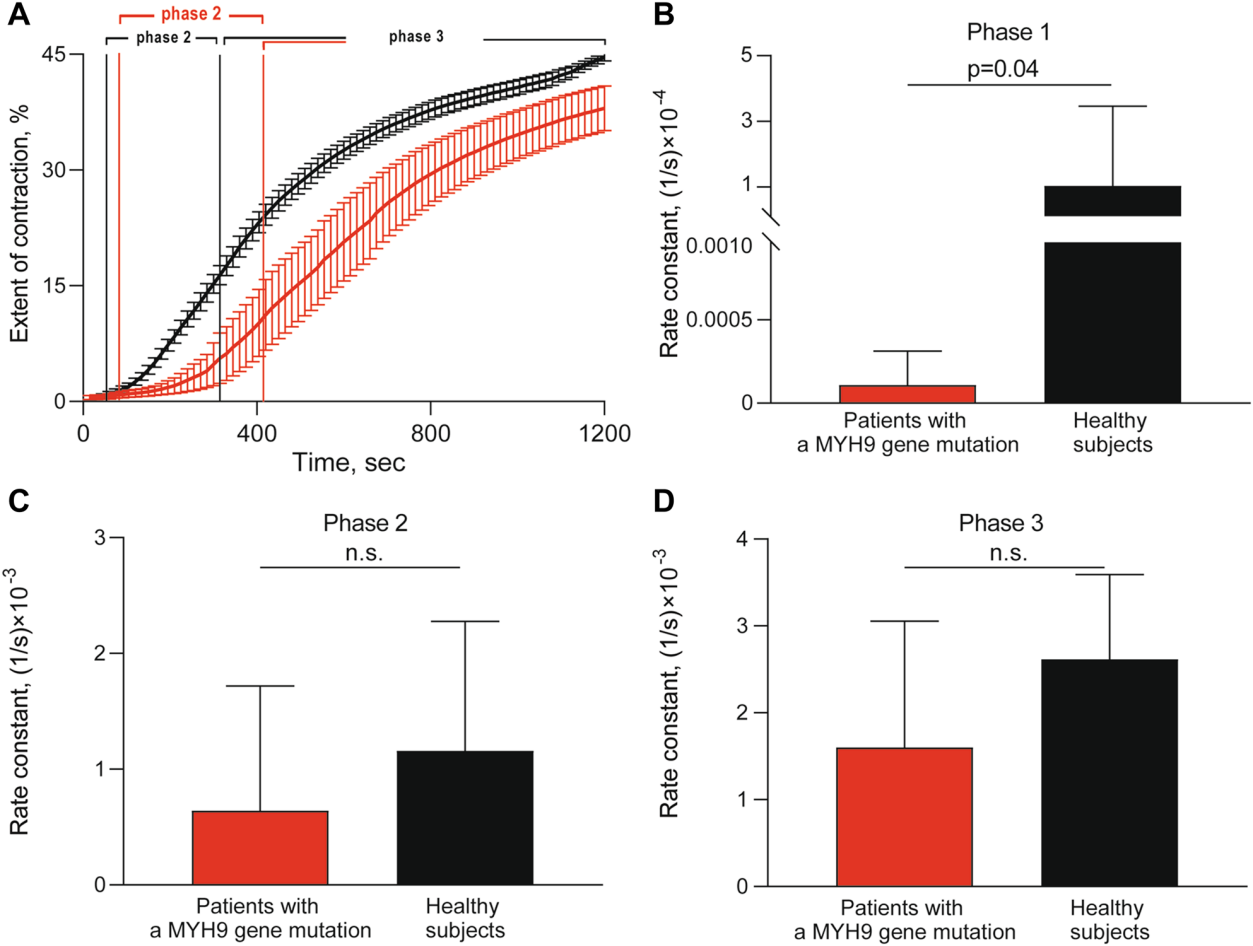
Comparative phase analysis of the averaged kinetic curves of clot contraction for patients with a *MYH9* gene mutation (red, n = 3) and healthy subjects (black, n = 15). On the averaged kinetic curves (**A**) transitions between the phases of contraction were determined by finding local maxima and minima within the instantaneous first derivatives. The curves were fit using a piecewise function, and the rate constant of each phase was determined. Average rate constants of phases 1, 2, and 3 (**B**,**C**,**D**, respectively) are shown for patients with a *MYH9* gene mutation (red) and healthy subjects (black). The results are presented as the median and interquartile range (25th and 75th percentiles). Mann–Whitney U test.

The ability of activated platelets to contract or shrink blood clots is an integral measure of platelet functionality. All parameters of the kinetics of clot contraction were impaired in proband M and her sister A, while in the proband’s mother F they were within normal ranges (Table 2). It has been previously shown that clot contraction occurs in three phases: initiation of contraction (phase 1), linear contraction (phase 2), and mechanical stabilization (phase 3)^19^. Regression analysis conducted on the averaged kinetic curves (Fig. 2A) revealed that in patients with the *MYH9* gene mutation, the rate constant of phase 1 was significantly reduced compared with healthy subjects, indicating impairment of the mechanisms of initiation of clot contraction (Fig. 2B). There were no significant differences in the kinetic rates of phases 2 and 3 between the patients and control healthy subjects (Fig. 2C,D). The duration of phase 1 was prolonged and the duration of phase 3 was shortened in patients with the *MYH9*-related disorder compared to healthy donors (Table 3). The routine hemostatic parameters studied in all the patients examined were within the reference values at the time of examination, while the stationary clot growth rate in the thrombodynamics assay was increased, indicating moderate chronometric hypercoagulability (Table 2).

**Table 2:** The extent of blood clot contraction, hemostasis and thrombodynamics in the patients with a *MYH9* gene mutation examined.

Parameters (in brackets—reference values)	Proband M	Sister of proband A	Mother of proband F
The final extent of blood clot contraction (42–49), %	**33%**	**39%**	42%
Lag period of clot contraction (75–255), sec	**360**	**405**	225
Area under the kinetic curve of clot contraction (256–393), a.u	**194**	**206**	310
Average velocity of clot contraction (0.034–0.041), %/sec	**0.027**	**0.032**	0.034
aPTT (25–36), sec	27.0	28.5	29.1
Prothrombin time (9.4–12.5), sec	11.1	11.2	10.2
INR (0.9–1.5)	0.95	0.96	**0.88**
Fibrinogen (1.8–3.5), g/l	3.2	**3.6**	2.9
Stationary clot growth rate in thrombodynamics (20–29), μM/min	**32.3**	**34.8**	**29.4**
Initial clot growth rate in thrombodynamics (38–56), μM/min	49.2	54.8	53.5
Maximum density of the clot in thrombodynamics (15,000–32,000), a. u	29,788	22,874	27,436

The numbers in bold are outside the corresponding reference range.

**Table 3:** Comparative phase analysis of the averaged kinetic curves of blood clot contraction for patients with a *MYH9* gene mutation and healthy subjects (control).

Phases of clot contraction	Patients with MYH9 gene mutation (n = 3)	Control group (n = 10)
**Phase rate constants**		
Phase 1, 1/s × 10^–4^	0.0001* (0.00005; 0.0003)	1.0 (0.04; 3.5)
Phase 2, 1/s × 10^–3^	0.6 (0.4; 1.7)	1.1 (0.6; 2.2)
Phase 3, 1/s × 10^–3^	1.6 (1.3; 3.1)	2.6 (1.6; 3.6)
**Phase durations**		
Phase 1, s	120** (110; 250)	70 (60; 80)
Phase 2, s	285 (195; 290)	155 (124; 243)
Phase 3, s	795* (660; 895)	985 (890; 1003)

The results are presented as the median and interquartile range (25th and 75th percentiles). *p < 0.05; **p < 0.01 compared to control. Mann–Whitney U test.

### Background functional state and reactivity of platelets

According to the flow cytometry data, all the patients examined displayed background or "spontaneous" platelet activation, which was revealed by 6–13-fold overexpression of P-selectin and active αIIbβ3 integrin compared to normal subjects in the absence of an activating stimulus (Table 4, Figs. S2 and S3). On the contrary, after addition of a potent activator (peptide TRAP-6) the proportion of platelets expressing the active αIIbβ3 integrin in the proband M and the proband’s sister was reduced substantially about 1.5-fold compared to the response to the activator observed in TRAP-stimulated platelets from healthy donors (Table 4). Expression of P-selectin on platelets in response to stimulation was also reduced in the sister of the proband compared to the control (Figs. S4 and S5). These results indicate a continuous background activation of circulating platelets associated with their refractoriness, i.e., reduced ability to respond to a physiological stimulus.

**Table 4:** Flow cytometry-measured expression of active integrin αIIbβ3 (mAb PAC-1 binding) and P-selectin ﻿in resting and stimulated platelets from the blood of patients with a *MYH9* gene mutation.

	Expression of active integrin αIIbβ3		Expression of P-selectin	
	Resting platelets	TRAP-stimulated platelets	Resting platelets	TRAP-stimulated platelets
Control heathy subjects (n = 10)	1.8% (1.2; 2.0)	92% (88; 92)	1.1% (0.6; 1.8)	79% (65; 81)
Proband M	**24%**	**49%**	**26%**	71%
Sister of proband A	**14%**	**62%**	**36%**	**64%**
Mother of proband F	**15%**	**82%**	**11%**	78%

(1) Results are presented as fractions of platelets expressing active integrin αIIbβ3 or P-selectin; (2) the results for control heathy subjects are presented as the median and interquartile range (25th and 75th percentiles); (3) the numbers in bold are outside the normal ranges determined in control healthy subjects.

### Shape and size of platelets studied with scanning electron microscopy

**Figure 3:**
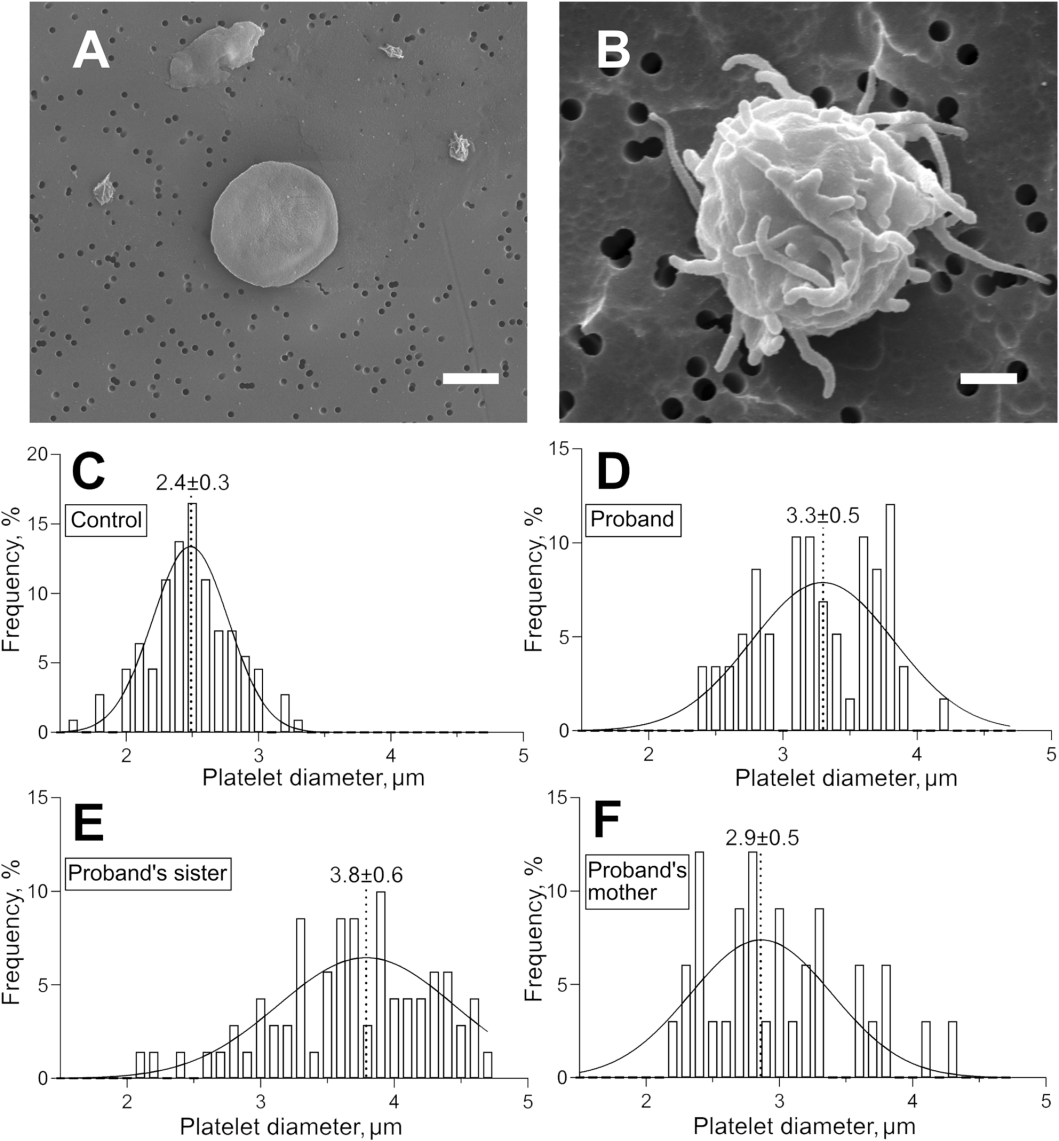
Characteristic scanning electron micrographs of platelets from a healthy donor (**A**) and the sister of proband with a *MYH9* gene mutation (**B**). In (**A**): the typical morphology of a normal resting platelet: normal size, discoid shape, absence of filopodia. In (**B**): a typical platelet from a patient with a *MYH9*-related disorder with morphological signs of activation: large size, loss of discoid shape, formation of multiple filopodia. Magnification bars = 1 µm. (**C**–**F**) Histograms of platelet diameters fitted with a Gaussian function (solid line) in a control healthy donor (**C**) and in the patients with a *MYH9* gene mutation (**D**–**F**). In (**C**–**F**) the Gaussian peak centroids and standard deviations are shown.

Detailed morphology of platelets from proband M, the sister of proband A, and the mother of proband F were studied with high-resolution scanning electron microscopy. In all the samples analyzed, platelets had heterogeneous morphology and could be segregated into two major types: (1) disc-shaped platelets without or with one or two short filopodia (corresponding to resting platelets) (Fig. 3A); (2) platelets that have lost their discoid shape and formed multiple filopodia (activated platelets) (Fig. 3B). Based on the morphological criteria, the shares of resting and activated platelets in the samples varied substantially. The smallest fraction of activated platelets among the patients was in the proband’s mother F (23%, n = 80), while in the proband M and the proband’s sister A, the fractions of activated platelets were much higher and reached 74% (n = 86) and 78% (n = 62), respectively (*p* < 0.0001, χ^2^-test). The proportions of activated platelets determined using the morphological criteria corresponded to the data obtained with flow cytometry (Table 4), both indicating an increased background platelet activation in the proband M and the proband’s sister A.

Since platelet macrocytosis, along with thrombocytopenia, is one of the characteristic features of the *MYH9-*related disorders, we used scanning electron microscopy to compare platelet size in the patients examined versus a healthy donor (Fig. 3). All three patients had platelets with an average diameter that was significantly larger than that of the control (2.4 ± 0.3 μm, n = 109; *p* < 0.0001 for all). The largest platelets were found in the sample of the proband’s sister A with an average diameter 3.7 ± 0.6 µm (n = 70); in the proband M, the average diameter was 3.2 ± 0.5 µm (n = 58), and in the proband’s mother F it was 2.9 ± 0.5 μm (n = 33). Thus, the results confirm the marked platelet macrocytosis in the patients with the *MYH9* gene mutation. Based on the platelet size distributions (Fig. 3C–F), we calculated manually the weighted mean platelet volume for each patient (Table 1) and for the normal control subject. Then, using the mean platelet volumes and individual platelet counts, we calculated the total platelet volume fraction or plateletcrit. Remarkably, despite the deep thrombocytopenia, the proband’s mother (0.13%) and the proband’s sister (0.15%) had plateletcrit values similar to that of the control subject (0.13%) and within the reported normal range (0.13–0.43%)^20^. The proband, who had an extremely low platelet count (36 × 10^9^/l), had the smallest plateletcrit (0.07%).

### Platelet ultrastructure

**Figure 4:**
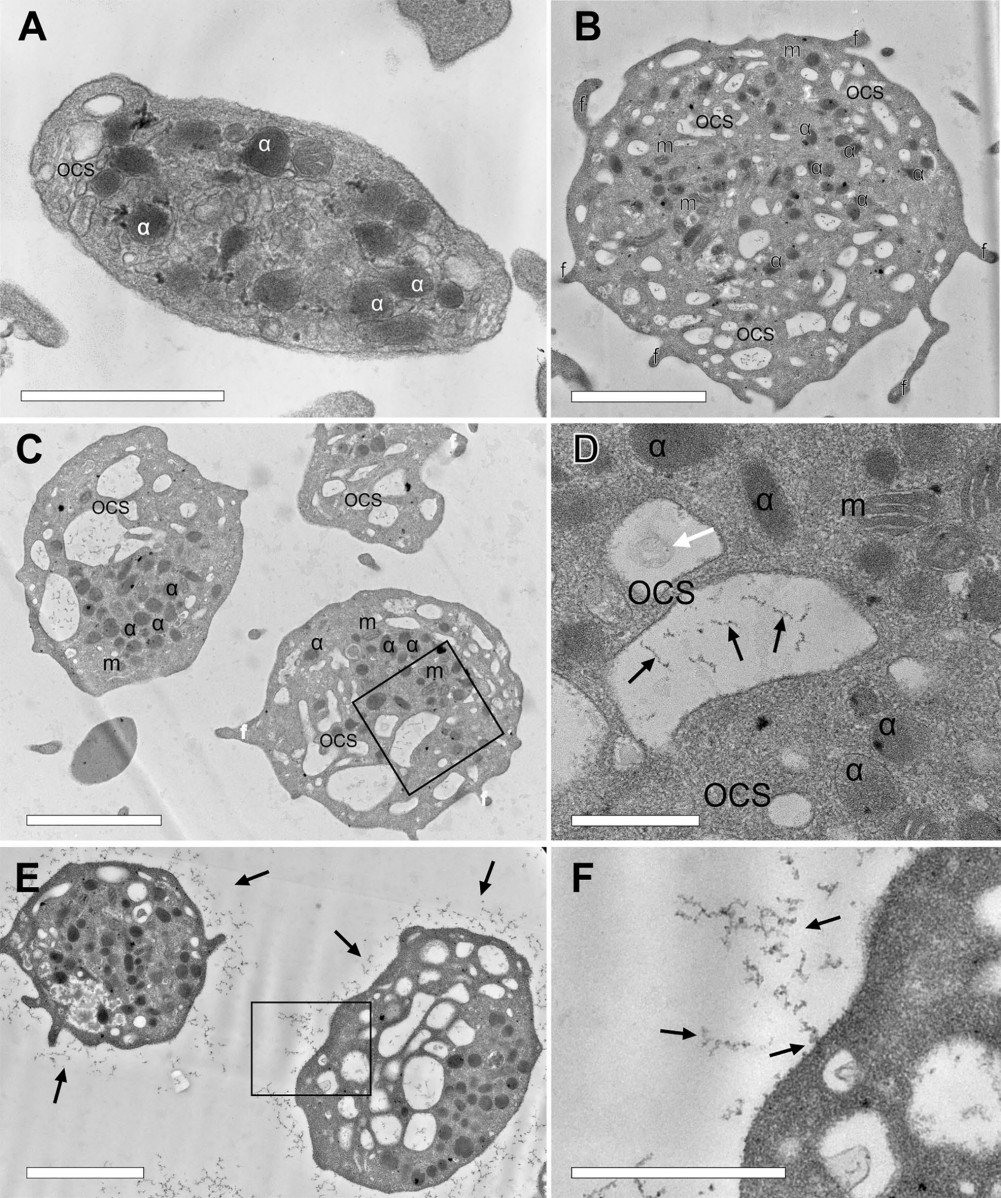
Representative transmission electron micrographs of platelets from a healthy donor (**A**) and the proband’s sister with a *MYH9* gene mutation (**B**–**F**). (**D**,**F**) are the zoomed-in areas marked with a rectangle in micrographs (**C**,**E**), respectively. Designations: *α*—alpha-granules; *OCS*—open canalicular system; *m*—mitochondria; *f*—filopodia. Black arrows in D-F indicate filamentous inclusions within OSC (**D**) and around platelets (**E**,**F**); the white arrow in D indicates a vesicular structure. Magnification bars: (**A**,**F**)—1 µm; (**B**,**C**,**E**)—2 µm, (**D**)—500 nm.

We studied the ultrastructure of platelets obtained from the blood of the proband’s sister A, because in this patient the functional (Tables 1, 2, 4) and morphological (Fig. 3B) alterations in platelets were the most pronounced. Expectedly, transmission electron microscopy confirmed (Fig. 4A,B) that the patient's platelets were larger (average diameter 3.4 ± 0.6 µm, n = 103) than platelets from a healthy donor (2.3 ± 0.5 µm, n = 29, *p* < 0.0001). Consistent with the images obtained using scanning electron microscopy, most of the patient's platelets had multiple membrane outgrowths (filopodia) extending from a rounded and large platelet body (Fig. 4B). However, the main ultrastructural feature of the patient's platelets were giant dilatations of the open canalicular system (Fig. 4C), which contained inclusions in the form of short filamentous structures and/or membrane vesicles (Fig. 4D). Similar fragmented filamentous structures were often observed in the extracellular space around platelets (Fig. 4E,F), so the broken filamentous structures within the dilated open canalicular system might comprise internalized particles, perhaps originating from disintegrated glycocalyx. Similar filamentous structures of uncertain source within the open canalicular system were observed earlier using transmission electron microscopy of platelets from a *MYH9* patient with the R1933X mutation^21^. The patient's platelet organelles were clearly visible (α-granules, mitochondria, open canalicular system), the integrity of the plasma membrane was not compromised, and the plasma membrane was smooth and easily discernible.

Although we did not see degranulation in the unstimulated platelets, we revealed a confluence or fusion of the α-granules with the open canalicular system (Fig. S6), which is a sign of the beginning of granular secretion, resulting in the release and overexpression of integrin αIIbβ3 and P-selectin revealed with flow cytometry. These findings may reflect elevated background platelet activation and secretion, as well as the observed differences between the levels of increased P-selectin in response to TRAP in platelets from healthy controls compared with patients.

## Discussion

One of the polypeptide chains of non-muscle myosin IIA is encoded by the *MYH9* gene; therefore, mutations in this gene are associated with alterations of non-muscle myosin IIA, the main generator of contractile force in many cell types, including platelets^5^. In megakaryocytes, mutations in the *MYH9* gene cause disturbance of thrombocytopoiesis and the development of macrothrombocytopenia^22,23^. From mouse models with a defect in the *MYH9* gene, it has been shown that most homozygous *MYH9* gene mutations are lethal and, therefore, the observed *MYH9* defects are heterozygous. It has also been found that the bleeding phenotype does not necessarily depend on the extent of thrombocytopenia and may be caused by inherent defects in platelet function, such as contractility and outside-in signaling associated with integrin β3 phosphorylation and accumulation of phosphatidylinositol(3,4)P(2) following stimulation^9,24^. Despite numerous studies, the mechanistic relationship between the genetic defects in the *MYH9* gene and the hemorrhagic phenotype of varying severity remains largely unclear, despite its practical and theoretical importance^25^.

49 different defects have been described in 12 exons of the *MYH9* gene^5^. The criteria for the differential diagnosis of disorders associated with *MYH9* gene mutations are summarized in Table S1. In the family case described here, the Fechtner and Epstein syndromes were excluded due to the absence of characteristic clinical manifestations, such as nephritis, deafness, and cataracts. In our case, the dual diagnosis of May-Hegglin anomaly/Sebastian syndrome was made due to the inability to differentiate these two closely related disorders. Potentially, they could be distinguished using transmission electron microscopy of leukocytes by differences in the diameter of Döhle-like bodies^3^, but all other genetic, laboratory, and clinical indicators of May-Hegglin anomaly and Sebastian syndrome are indistinguishable^11^.

In this study, three female family members with a documented defect in the *MYH9* gene had a history of heavy menstruation, spontaneous skin ecchymosis, and postpartum hemorrhage (in the proband’s sister), although there were no visible signs of bleeding at the time of examination, despite moderate to severe thrombocytopenia. This is consistent with the known predisposition of patients with *MYH9* gene mutations to hemorrhage, albeit the manifestations may vary from asymptomatic to severe bleeding^2,11^. The main laboratory symptom in all the patients examined was thrombocytopenia of varying severity (Table 1), which led to the erroneous initial diagnosis of immune thrombocytopenic purpura. A hematologist expressed doubt about the correctness of this diagnosis based on a family history of thrombocytopenia and a marked increase in the immature platelet fraction in the proband, her sister and mother. Microscopy of the blood smears revealed large platelets and Döhle-like bodies in leukocytes, which justified a putative diagnosis of either May-Hegglin anomaly or Sebastian syndrome in the proband, her sister, and mother. This diagnosis was verified by a genetic analysis of all three patients that revealed a heterozygous C5797T mutation in the 41st exon of the *MYH9* gene, which corresponds to the R1933X mutation in the heavy chain of myosin IIA. This mutation was first identified in 2000 in the members of an Italian family with clinical manifestations of the disorder previously known as May-Hegglin anomaly or Sebastian syndrome^26^.

Since the *MYH9* gene encodes myosin IIA, which interacts with actin to form the force-generating machinery, mice with *MYH9* mutations have inherently impaired contractility, determined both in vitro^24,27,28^ and in vivo^9,24,29^. The impaired platelet contractility results in reduced compaction of blood clots, which is necessary for efficient hemostasis^30^. However, it is not clear in either mice or humans with *MYH9*-related hemostatic disorders if the reduced clot contraction is due to thrombocytopenia or impaired platelet contractility or both. Our data show a moderate decrease in the degree of contraction of blood clots in patients with *MYH9* gene mutation (Table 2). To distinguish between these possibilities, we determined the total platelet volume (plateletcrit) in the patients (Table 1). Surprisingly, in two of the three patients examined with the weakest hemorrhagic phenotypes, the total platelet volume was within the normal range. Notably, the normal plateletcrit was observed despite thrombocytopenia and attributed to the significant fraction of huge platelets. It suggests that the observed defect in platelet contractility is likely due to qualitative platelet deficiencies rather than the low platelet counts. From a more general standpoint, it is possible that the increased platelet size (and related contractile potential of individual giant platelets) comprise a compensatory hemostatic mechanism that helps to overcome a low platelet count and reduce the clinical expression of the bleeding phenotype. Additional strong arguments that the impaired contraction of blood clots in the *MYH9-*related disorders is due to platelet dysfunction are based on the distinct qualitative changes in platelets, both functional (Tables 2 and 4) and structural (Figs. 1, 3, 4). Regardless of the underlying mechanisms, the suppressed compaction of blood clots may be an important pathogenic mechanism for hemostatic disorders with a bleeding tendency in diseases associated with the *MYH9* gene mutation.

One of the most interesting findings is that platelets from the patients examined are in a pre-activated and possibly exhausted state, which contributes to the bleeding diathesis. In our study, flow cytometry revealed a background activation of unstimulated platelets in the proband and the proband’s sister, assessed by overexpression of P-selectin and active αIIbβ3 integrin (Table 4). The cause of this chronic platelet hyperactivation in the bloodstream may be due to hypercoagulability and enhanced thrombin generation associated with thrombocytopenia^31^. In line with these observations, our study revealed moderate chronometric hypercoagulability in all the patients examined (Table 2), which may comprise a compensatory hemostatic mechanism in response to thrombocytopenia.

After stimulation of PAR1 receptors, the fraction of P-selectin-expressing platelets in these patients was reduced compared to healthy donors, which indicates reduced platelet reactivity (Table 4). This partial platelet refractoriness may be another mechanism for the inefficiency of the platelet hemostasis and for the hemorrhagic tendency in the *MYH9* gene mutations. In addition to the storage pool deficiency and partial degranulation, a plausible mechanism for this dysfunction is shedding of the membrane adhesive proteins along with fragments of glycocalyx observed around platelets (Fig. 4E,F). Taken together, these data strongly suggest that thrombocytopathy causes hemostatic abnormalities, aggravating the bleeding phenotype due to thrombocytopenia in *MYH9*-related disorders. It should be noted that a similar chronic platelet hyperactivation followed by platelet exhaustion and secondary dysfunction, including reduced reactivity and contractility, were revealed in a number of (pro)thrombotic conditions^32–38^. The search for fundamental causes and mechanisms of the impaired contraction of blood clots in (pro)thrombotic conditions has led to the discovery of a common mechanism. The impaired contraction of blood clots in thrombotic conditions turned out to be a consequence of chronic, continuous activation of platelets in the blood stream, leading to their secondary refractoriness and dysfunction, including impaired contractility. The mechanisms of platelet dysfunction may be related to energetic exhaustion and ATP depletion^39,40^, storage pool deficiency^41,42^ as well as shedding or cleavage of surface receptors^43–45^. The phenomenon of platelet exhaustion following chronic activation newly revealed in the patient with the *MYH9* gene mutation has been observed in pathological conditions of various etiologies^33,36,37^. This the first instance that this biphasic platelet behavior has been shown in a bleeding disorder. This surprising similarity in the biphasic platelet behavior between (pro)thrombotic and hemorrhagic conditions reflects the multifaceted pathophysiological role of impaired platelet contractility: depending on the circumstances, it can promote thrombosis or enhance bleeding. In thrombotic patients, defective clot contraction causes a looser and more obstructive thrombus, while in bleeding disorders, poor clot contraction leads to a looser and more permeable (and therefore hemostatically less effective) clot.

There is an apparent controversy between the hemostatic effects of hypercoagulability and continuous platelet activation, causing secondary platelet dysfunction that contributes to the bleeding phenotype. We think that there is vicious circle comprising the following sequence of events. The primary impetus may be thrombocytopenia that is known to be often associated with hypercoagulability, which has an adaptive or compensatory nature^31^. The mechanism of this feedback is likely related to massive death (apoptosis?) of platelets and formation of procoagulant extracellular microparticles, either extosomes or exosomes^46^. The resulting hypercoagulability may have a dual effect: it can improve hemostasis but at the same time stimulate circulating platelets, causing or exaggerating their dysfunction, which we have revealed in the patients examined.

In summary, the reduced clot contraction in the *MYH9*-related disorder is the additive effect of two mechanisms: (i) defective platelet contractile machinery due to mutated non-muscle myosin IIA and (ii) secondary platelet refractoriness to stimulation due to preactivation. It is hard to assess the relative contribution of these two mechanisms and whether one promotes the other, but the refractoriness to thrombin is certainly a major factor leading to the impaired platelet contractility, especially in heterozygous mutations of the *MYH9* gene. The normal platelet response to TRAP of the mother’s platelets and her low fraction of activated platelets in combination with her normal clot contraction comprise a strong argument supporting the role of platelet refractoriness in suppressed clot contraction. It is noteworthy that the defective non-muscle myosin IIA has been a relatively obvious and well-known cause of reduced platelet contractility, while the platelet refractoriness due to preactivation is a hitherto unknown mechanism of platelet dysfunction, at least for the *MYH9*-related disorder studied here.

Taken together, the results of this study shed light on the importance of functional and morphological changes in platelets associated with mutation of the *MYH9* gene. These multiple qualitative platelet abnormalities, in combination with thrombocytopenia, contribute to bleeding risk in the *MYH9*-related disorder.

## Conclusions

This familial case study was aimed at the elucidating qualitative functional and structural alterations of platelets in a hereditary *MYH9*-related disorder known as the May-Hegglin anomaly or the Sebastian syndrome and characterized by macrothrombocytopenia associated with a tendency for bleeding. It has been shown that in patients with a heterozygous mutation C5797T (R1933X) in the *MYH9* gene, hemorrhagic manifestations of varying severity are due not only to thrombocytopenia but also to qualitative structural and functional defects in platelets. These defects show up as background platelet activation, which is combined paradoxically with partial platelet refractoriness, delayed initiation of clot contraction and contractile dysfunction, all of which can contribute to the formation of imperfect hemostatic clots and bleeding. At the same time, the bleeding phenotype may be reduced in this mutation due to platelet macrocytosis that compensates for thrombocytopenia by maintaining the total platelet volume (plateletcrit) and procoagulant potential sufficient for moderate systemic hypercoagulability and effective hemostasis. Altogether, the results obtained clearly show that the *MYH9*-related disorder studied is accompanied by multiple functional and structural platelet abnormalities that largely underlie the bleeding phenotypic manifestations.

## Methods

### Clinical material

Complete examination with genetic analysis included a patient M (proband, 35 y.o.), her sister A (38 y.o.) and their mother F (62 y.o.) (M, A, and F are initials of the patients’ first names). In addition, blood tests were performed in the proband’s grandmother (84 y.o.), the niece (2 y.o.), and the newborn daughter. The study was approved by the Ethics Committee of the Kazan Federal University (protocol #3 as of March 23, 2017). All experiments were performed in accordance with the approved guidelines. All the patients signed informed consent documents.

### Blood and its components

Venous blood was stabilized with 3.8% sodium citrate at a ratio of 9:1 by volume. Platelet-rich plasma (PRP) was obtained by centrifugation of citrated blood at 200 g for 10 min; platelet-free plasma was obtained by successive double centrifugation of PRP at 1600 g, 15 min, and at 10,000*g*, 5 min. An additional sample of venous blood was stabilized with K_3_-EDTA (final concentration 1.6 mg/ml) and used for hematological analyzes and genetic studies. Blood for analysis was used no later than 4 h after collection.

### Microscopy of blood smears, hematological and hemostatic tests

Whole capillary blood from a finger prick was mixed with a 3% EDTA solution at a 1:3 ratio. A drop of blood was transferred onto a glass slide, smeared, dried, and fixed in methanol for 10 min. A smear of peripheral blood was stained according to Romanowsky-Giemsa and analyzed in 10 microscopic fields at a 400 × optical magnification. Blood count was performed using an automatic hematological analyzer (Sysmex XN, Japan). Hemostasis was assessed with an automated coagulometer ACL TOP 500 (Instrumentation Laboratory, USA). The kinetics of directed clot formation in blood plasma (thrombodynamics assay) was studied as described previously^47^.

### Contraction of blood clots

The kinetics and extent of platelet-driven blood clot contraction were quantified based on the dynamic registration of the size of a thrombin-induced blood clot during shrinkage^19^. Citrated blood samples activated by 1 U/ml thrombin and 2 mM CaCl_2_ were transferred to a transparent plastic cuvette that was pre-lubricated with 4 v/v% Triton X-100 in 150 mM NaCl to prevent sticking of the clot to the walls of the chamber. Using light-scatter-based tracking, changes in the clot size during contraction were measured every 15 s over 20 min. Serial images of the shrinking clot were converted into a kinetic curve of clot contraction, from which the following parameters were extracted: (1) the extent of contraction, i.e. the degree of shrinkage of the clot after 20 min relative to its initial size; (2) lag time, i.e. the time from the addition of thrombin until the clot reaches 95% of its initial size; (3) the area under the kinetic curve, which reflects the amount of mechanical work on clot compression; (4) the average contraction velocity is the extent of clot contraction divided by the time of contraction (20 min).

### Flow cytometry of platelets

The functional state of platelets in PRP was assessed by flow cytometry before and after activation with the thrombin-receptor activating hexapeptide (TRAP-6) by expression of P-selectin (assessed by the binding of anti-CD62p antibodies) and active αIIbβ3 integrin (reacting with PAC-1 antibodies). TRAP-6 (50 μM final concentration as in^48^) was added to PRP and incubated for 3 min at room temperature. After addition of fluorescently labelled antibodies (followed by incubation for 10 min at room temperature), platelets were analyzed using a FacsCalibur flow cytometer equipped with a BD CellQuest software. The platelet gate was determined based on their size (forward scatter, FSC) and granularity (side scatter, SSC); 5000 platelets were counted in each sample. The FlowJo X software was used for data analysis.

### Scanning electron microscopy of platelets

For scanning electron microscopy of platelets, PRP was diluted tenfold with Tyrode's buffer (4 mM HEPES; 135 mM NaCl; 2.7 mM KCl; 2.4 mM MgCl_2_; 3.3 mM NaH_2_PO_4_; 5.6 mM D-glucose; pH 7.4), after which it was fixed with glutaraldehyde at a final concentration of 2.5% and deposited on a polycarbonate filter (0.1 or 0.4 μm pore size) by centrifugation at 500 g, 5 min, with fast acceleration and slow deceleration. The precipitated platelets were washed with phosphate buffer (0.1 M NaH_2_PO_4_, 0.1 M Na_2_HPO_4_, pH 7.2), then they were dehydrated with ascending concentrations of ethanol and incubated in hexamethyldisilazane followed by drying. Dehydrated samples were sputter-coated with a thin layer of gold–palladium on Polaron e5100 or Quorum Q150T instruments and used for scanning electron microscopy (Quanta 250 FEG microscope, ThermoFisher Scientific).

### Transmission electron microscopy of platelets

For transmission electron microscopy of platelets, PRP was diluted 10 times with Tyrode's buffer, after which it was fixed with 2.5% glutaraldehyde, washed with phosphate buffer (0.1 M NaH_2_PO_4_, 0.1 M Na_2_HPO_4_, pH 7.2), and post-fixed with 1% OsO_4_ in the presence of sucrose (25 mg/ml). Next, the samples were dehydrated with ethanol solutions in ascending concentrations, after which the samples were treated with acetone and propylene oxide. Dehydrated samples were impregnated sequentially, with 12–24-h intervals at 4 °C, in a mixture of epoxy resin (Epon-812) and propylene oxide mixed at the ratios of 1:2, 1:1 and 2:1. After that, the samples were embedded in pure epoxy resin and left to polymerize in a thermostatic chamber at 37 °C. Every 12–24 h, the temperature was raised from 37 °C to 45 °C, then to 60 °C. Sections were obtained using a Leica UC7 ultramicrotome (Germany), and they were counterstained with saturated aqueous uranyl acetate and 0.4% aqueous lead citrate. The preparations were examined using a Hitachi HT7700 Exalens electron microscope (Japan).

### Molecular genetic analysis

DNA extraction to detect mutations in the *MYH9* gene was performed from the buccal epithelium using the DNA-Express reagent kit (SPF Litekh, Russia) according to the manufacturer's instructions. The presence or absence of potential mutations in exons 26 (T1155I) and 27 (R1165C) of the gene was determined with the qPCR method based on the TaqMan technology using commercial reagent kits from ThermoFisher Scientific (USA) (cat. no. C_27860266_10 and C_27860265_10, respectively). Mutations in other exons were detected by sequencing using primers selected with the CloneManager program (Table S2). The sequencing reaction was carried out using the BigDye Terminator v3.1 Cycle Sequencing Kit (Applied BioSystems, USA) followed by separation of the sequencing products on an ABI PRISM 3730 capillary analyzer (Applied BioSystems, USA). The sequencing results were analyzed using the FinchTV program followed by alignment of the *MYH9* gene exon sequences using the BLAST (https://blast.ncbi.nlm.nih.gov) and Mafft (https://mafft.cbrc.jp/alignment/server) software.

### Statistical analysis

GraphPad Prism 8 software package was used for statistical analysis. Pairwise comparisons were made using the Mann–Whitney test for non-parametric data and Student's *t*-test for parametric data. For multivariate analysis, the Kruskal–Wallis test followed by Dunn's test for non-parametric parameters and the one-way ANOVA test followed by Dukey's test for parametric data were used. Correlation analysis was carried out by the Spearman’s method. The χ^2^-test was used to analyze categorical parameters. The level of statistical significance was 95% (*p* < 0.05).

## Supplementary Information

Supplementary Information.

## Data Availability

All data generated or analyzed during this study are included in this published article and its Supplementary Information files.
