# Supplementary material for: A familial case of MYH9 gene mutation associated with multiple functional and structural platelet abnormalities
Source: Sci Rep. 2022 Nov 20;12:19975. doi: 10.1038/s41598-022-24098-5 (PMC9676191; doi:10.1038/s41598-022-24098-5)
Supplement: Supplementary file 1 — Supplementary Information. [file 41598_2022_24098_MOESM1_ESM.pdf]

Supplementary Information

A Familial Case of *MYH9* Gene Mutation Associated with Multiple Functional and Structural Platelet Abnormalities

Svetlana I. Safiullina[1]*, Natalia G. Evtugina[1]*, Izabella A. Andrianova[1], Rafael R. Khismatullin[1], Olga A. Kravtsova[1], Alina I. Khabirova[1], Chandrasekaran Nagaswami[2], Amina G. Daminova[1], Alina D. Peshkova[1], Rustem I. Litvinov[2], John W. Weisel[2]**

[1]Institute of Fundamental Medicine and Biology, Kazan Federal University, Kazan, Russian Federation

[2]Department of Cell and Developmental Biology, University of Pennsylvania, Philadelphia, United States

*Co-first authors, contributed equally

**Correspondence: Prof. John Weisel
University of Pennsylvania School of Medicine
421 Curie Blvd., BRB II/III, Room 1154
Philadelphia, PA 19104-6058 USA
Tel.: +1-215-898-3573
weisel@pennmedicine.upenn.edu

Table S1. Differential clinical and laboratory features of various macrothrombocytopenias associated with defects in the *MYH9* gene

Diseases	*Macro-thrombo-cytopenia*	*Inclusions in leukocytes (Döhle bodies)*	*Nephritis*	*Deafness*	*Cataract*	*Mutations of the MYH9 gene (myosin IIA heavy chain)*
May-Hegglin anomaly ICD-10 code D72.0	+	+	-	-	-	R1933X<br>T1155I<br>D1424N<br>E1841K<br>R1165C<br>5779delC
Sebastian Syndrome	+	+	-	-	-	T1155I<br>D1424N<br>E1841K<br>R1933X<br>R1165C<br>5779delC
Fechtner syndrome	+	+	+	+	+	R702C<br>D1424N<br>E1841K<br>R1933X
Epstein syndrome	+	-	+	+	-	R702C

Compiled from *Heath et al.* Am. J. Hum. Genet. 2001; 69(5):1033-1045

Table S2. Primer sequences for sequencing exons of the *MYH9* gene

Exon	*Potential mutation*	*Primer sequence*	*Amplifier size, b.p.*
2	N93R	F: 5'-CCAACTTGTTTTTCAGTGATGCACATC-3'<br>R: 5' - ACTCCTTCAAGCCCCCTTCTCAAC-3'	675
11	K371N	F: 5'-GTGGCTTTCTTCCTCTCTGCTCC-3'<br>R: 5'-ATTGTGCAAGAACCGTACTCAGGTC-3'	367
17	R702C	F: 5'-TCCAAACCTGTGGGCTGTAGCG-3'<br>R: 5'-ACTCAGTTCTACATGGATGGAGGAC-3'	384
31	D1424N	F: 5'-CATAACTGGGCAGATCCCTGGTG-3'<br>R: 5'-CTCTAAGCACTGGCCCCGCAC-3'	513
39	E1841K	F: 5'-AGCAAAGGCCTCCCTACGTGATC-3'<br>R: 5'-GCAGTCCTTTCTTGGTGACATTCG-3'	668
41	R1933X	F: 5'-GCTGTGGCTCCCAAGACTCC-3'<br>R: 5'-GGCAGGAGGAGGCATGTTCAC-3'	1722

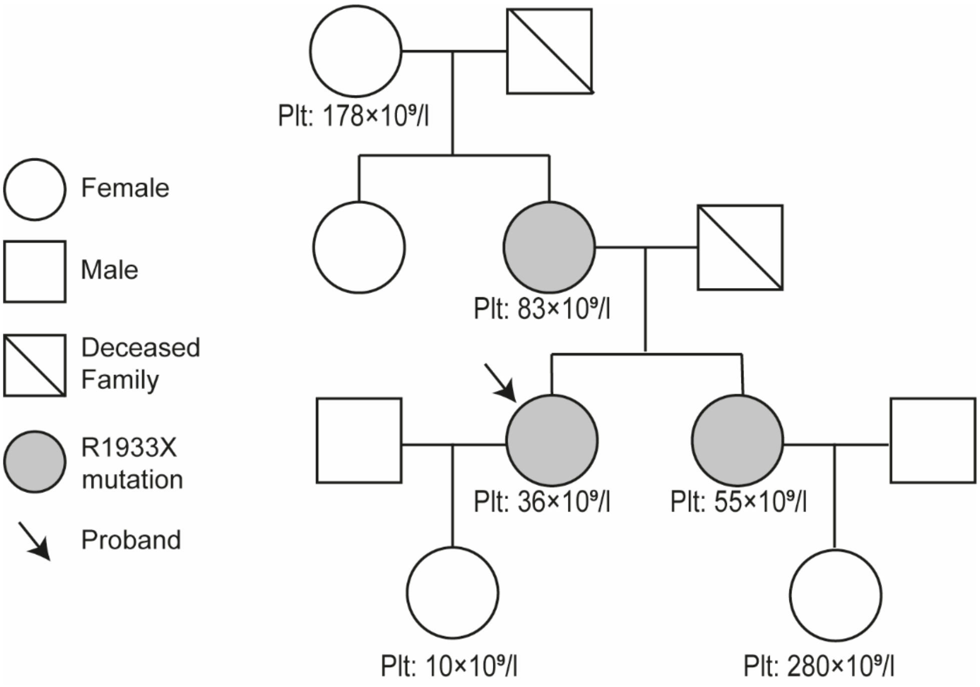

Figure S1. Pedigree of the patients with a *MYH9* gene mutation examined and platelet counts in the blood of family members at the time of examination.

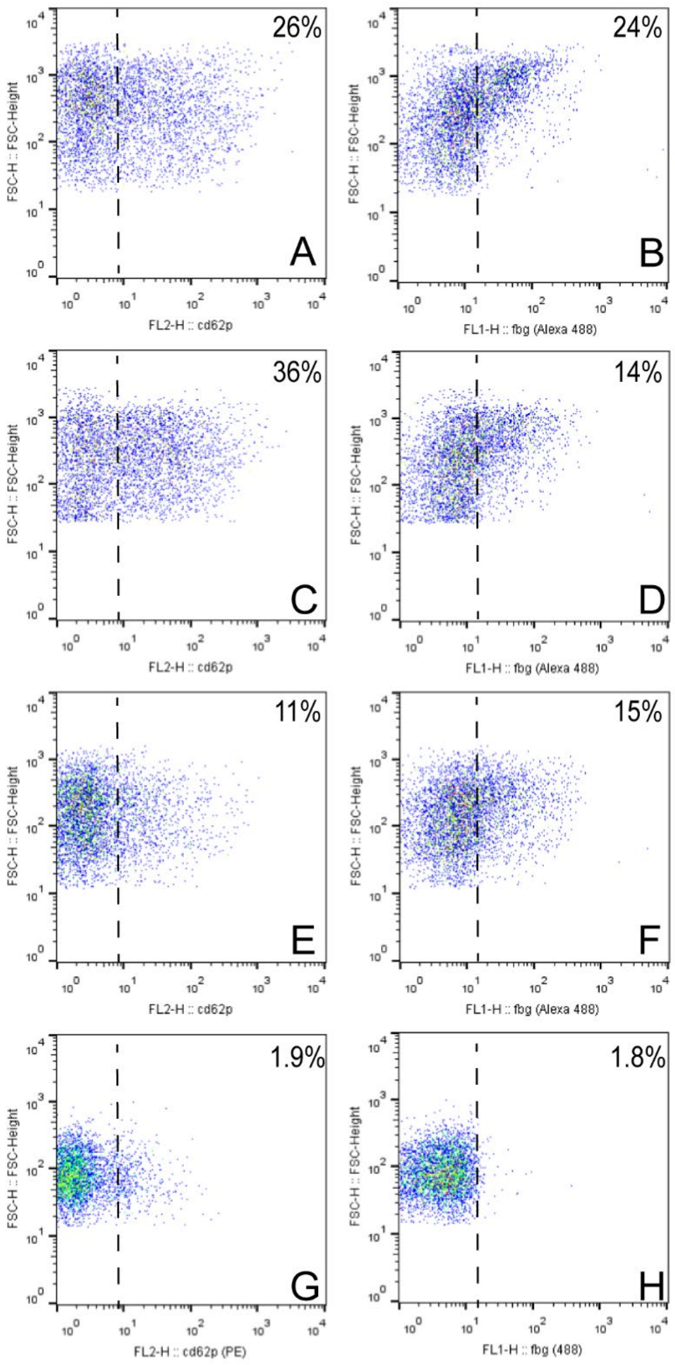

Figure S2. Dot plots showing overexpression of P-selectin (**A, C, E**) and the active integrin αIIbβ3 (**B, D, F**) in unstimulated platelets of the proband (A, B), proband's sister (C, D), and mother (E, F) compared to the control sample from a healthy subject (**G, H**). The results are shown as histograms in Figure S3.

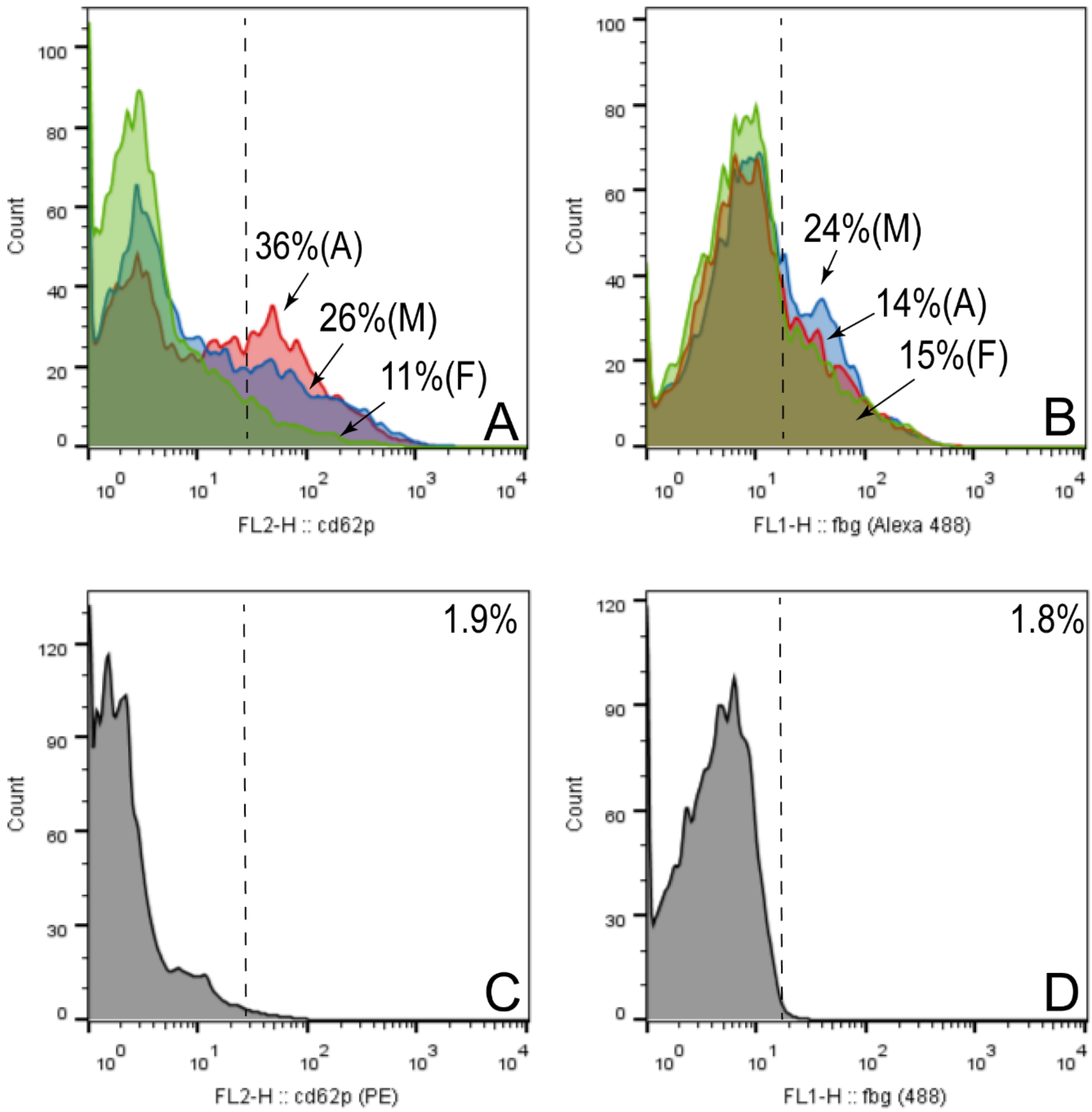

Figure S3. Histograms showing overexpression of P-selectin (**A**) and the active integrin αIIbβ3 (**B**) in unstimulated platelets of the three patients with the May-Hegglin anomaly compared to the control sample from a healthy subject (**C, D**). The results are shown as raw dot plots in Figure S2.

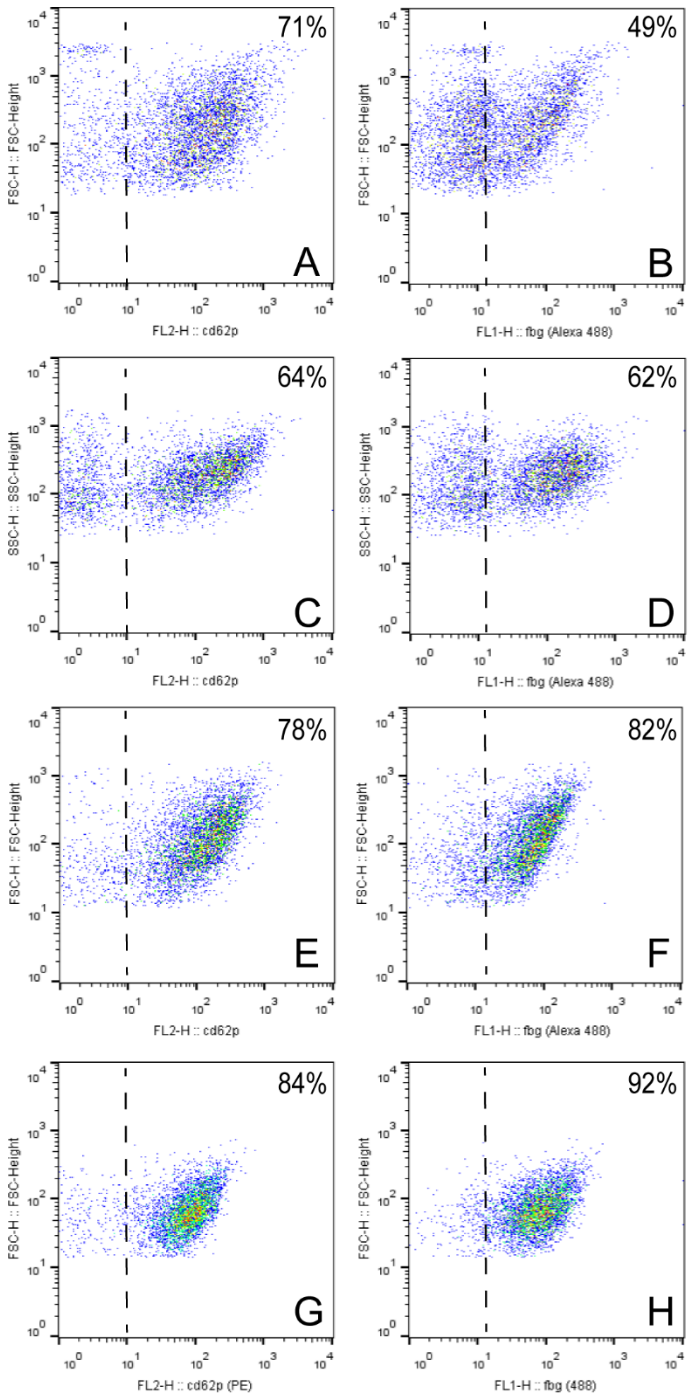

Figure S4. Dot plots showing reduced expression of P-selectin (**A, C, E**) and the active integrin αIIbβ3 (**B, D, F**) in TRAP-stimulated platelets of the proband (A, B), proband's sister (C, D), and mother (E, F) compared to the control sample from a healthy subject (**G, H**). The results are shown as histograms in Figure S5.

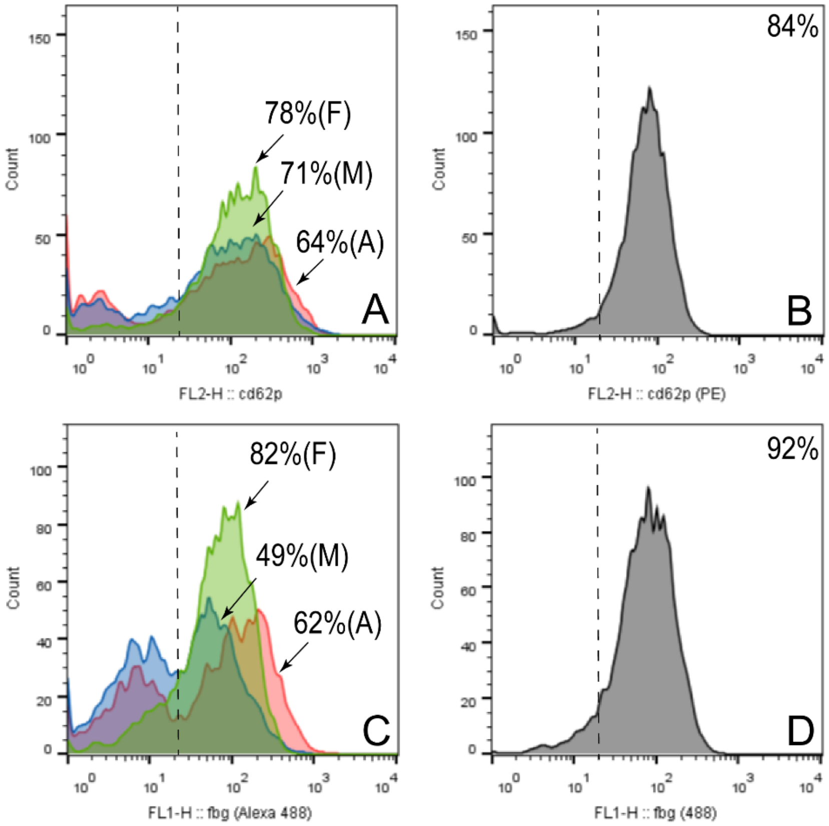

Figure S5. Histograms showing reduced expression of P-selectin (**A**) and the active integrin αIIbβ3 (**C**) in TRAP-stimulated platelets of the three patients with the May-Hegglin anomaly compared to the control sample from a healthy subject (**B, D**). The results are shown as raw dot plots in Figure S4.

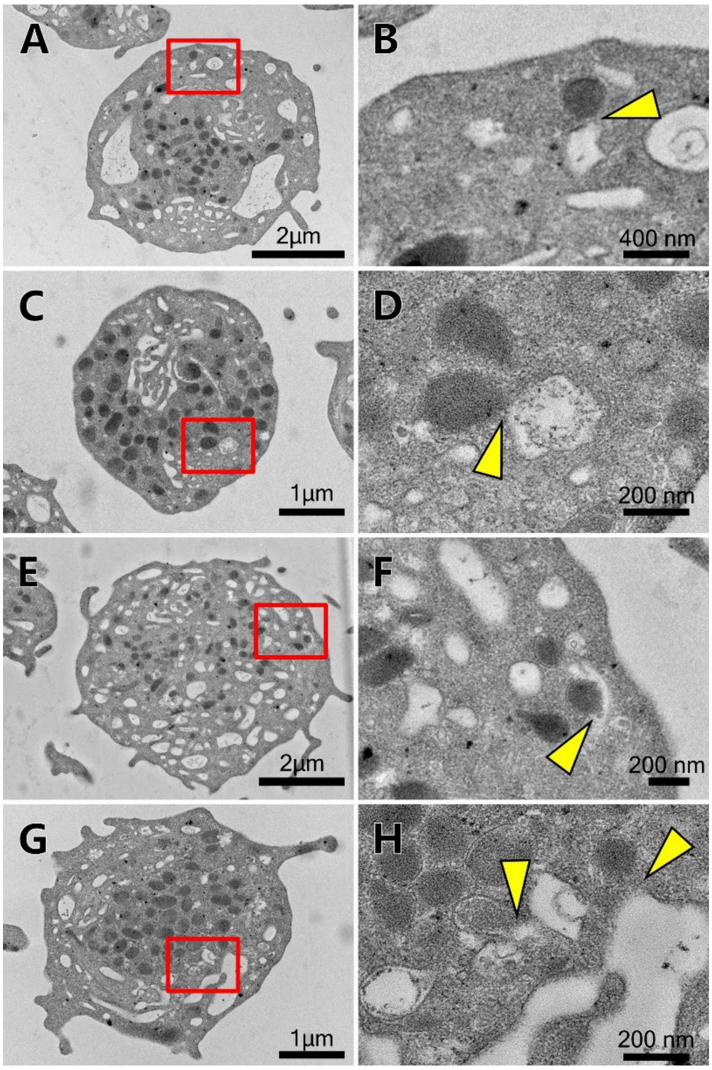

Figure S6. Representative TEM images of individual platelets from the proband's sister with the *MYH9* gene mutation. Images in **A, C, E** and **G** have the areas marked with red rectangles that are zoomed-in and shown in **B, D, F** and **H**, respectively. Yellow arrowheads indicate the points of confluence (fusion) of the α-granules with dilated OCS.
